# Plasmonic Hot-Electron Reactive Oxygen Species Generation: Fundamentals for Redox Biology

**DOI:** 10.3389/fchem.2020.591325

**Published:** 2020-12-03

**Authors:** Elisa Carrasco, Juan Carlos Stockert, Ángeles Juarranz, Alfonso Blázquez-Castro

**Affiliations:** ^1^Department of Biology, Faculty of Sciences, Autonomous University of Madrid, Madrid, Spain; ^2^Area Investigación, Instituto de Oncología “Angel H. Roffo”, Universidad de Buenos Aires, Buenos Aires, Argentina; ^3^Cátedra de Histología y Embriología, Instituto de Investigación y Tecnología en Reproducción Animal, Facultad de Ciencias Veterinarias, Universidad de Buenos Aires, Buenos Aires, Argentina

**Keywords:** plasmon, hot-electron, metal nanoparticle, reactive oxygen species, redox biology, singlet oxygen, photodynamic therapy

## Abstract

For decades, the possibility to generate Reactive Oxygen Species (ROS) in biological systems through the use of light was mainly restricted to the photodynamic effect: the photoexcitation of molecules which then engage in charge- or energy-transfer to molecular oxygen (O_2_) to initiate ROS production. However, the classical photodynamic approach presents drawbacks, like *per se* chemical reactivity of the photosensitizing agent or fast molecular photobleaching due to *in situ* ROS generation, to name a few. Recently, a new approach, which promises many advantages, has entered the scene: plasmon-driven hot-electron chemistry. The effect takes advantage of the photoexcitation of plasmonic resonances in metal nanoparticles to induce a new cohort of photochemical and redox reactions. These metal photo-transducers are considered chemically inert and can undergo billions of photoexcitation rounds without bleaching or suffering significant oxidative alterations. Also, their optimal absorption band can be shape- and size-tailored in order to match any of the near infrared (NIR) biological windows, where undesired absorption/scattering are minimal. In this mini review, the basic mechanisms and principal benefits of this light-driven approach to generate ROS will be discussed. Additionally, some significant experiments *in vitro* and *in vivo* will be presented, and tentative new avenues for further research will be advanced.

## Introduction

Redox biology and redox control of biological functions are fundamental aspects of cell biology. It is a relatively young field, but its mechanics and ramifications are extremely important for all cellular processes: cell proliferation, survival, migration, differentiation, programmed cell death, organogenesis, immunology, aging, cancer, and oncotherapy, etc. (Sies, 2020). Advancement in this emerging field critically depends on the controlled production of reactive oxygen species (ROS), to understand how redox signaling modulates biological functions (Zhang et al., 2019a). A classical approach to this has been the use of the photodynamic effect to induce ROS production (Macia and Heyne, 2015). This approach has been in use for oncological treatments for several decades under the name of photodynamic therapy (PDT). Although PDT is well-established as a therapeutic treatment, there are serious disadvantages that still jeopardize the modality (Sorrin et al., 2020). Indeed, low actinic light penetration is cited as one of the severest (Fan et al., 2016). Moving into the near-infrared (NIR, 700–1,100 nm) biological window to photoexcite compounds is a very sought-after strategy (Deng et al., 2017). Introduction of new photosensitizing compounds, like metal nanoparticles, is another promising front (Chen et al., 2020).

Metal nanoparticles, particularly gold, silver or palladium, provide many advantages because they absorb in the NIR and have been shown to produce ROS upon illumination (Protti et al., 2017). The mechanism producing ROS in illuminated metal nanoparticles is the generation of energetic hot-electrons due to the plasmonic effect, which appears in metals as a consequence of their particular electronic structure (Halas, 2019). These nanoparticles display chemical non-reactivity in the darkness and a resistance to oxidation that makes them ideal photosensitizing elements in PDT (Chen et al., 2020). These advantages can be exploited in the field of redox biology research, as metal nanoparticles are excellent vehicles for controlled photogeneration of ROS. Indeed, they are being employed for light-driven environmental remediation that degrades pollutants by ROS (Wang et al., 2018). This mini-review will introduce the fundamentals of the plasmonic effect and its potential and realized application in redox biology.

## Plasmonic Hot-Electrons

The mechanism producing hot-electrons in metal nanoparticles, which ultimately will lead to ROS generation, is different from the photodynamic effect commonly employed in PDT. This mechanism is singular and will be discussed in what follows.

**Figure 1:**
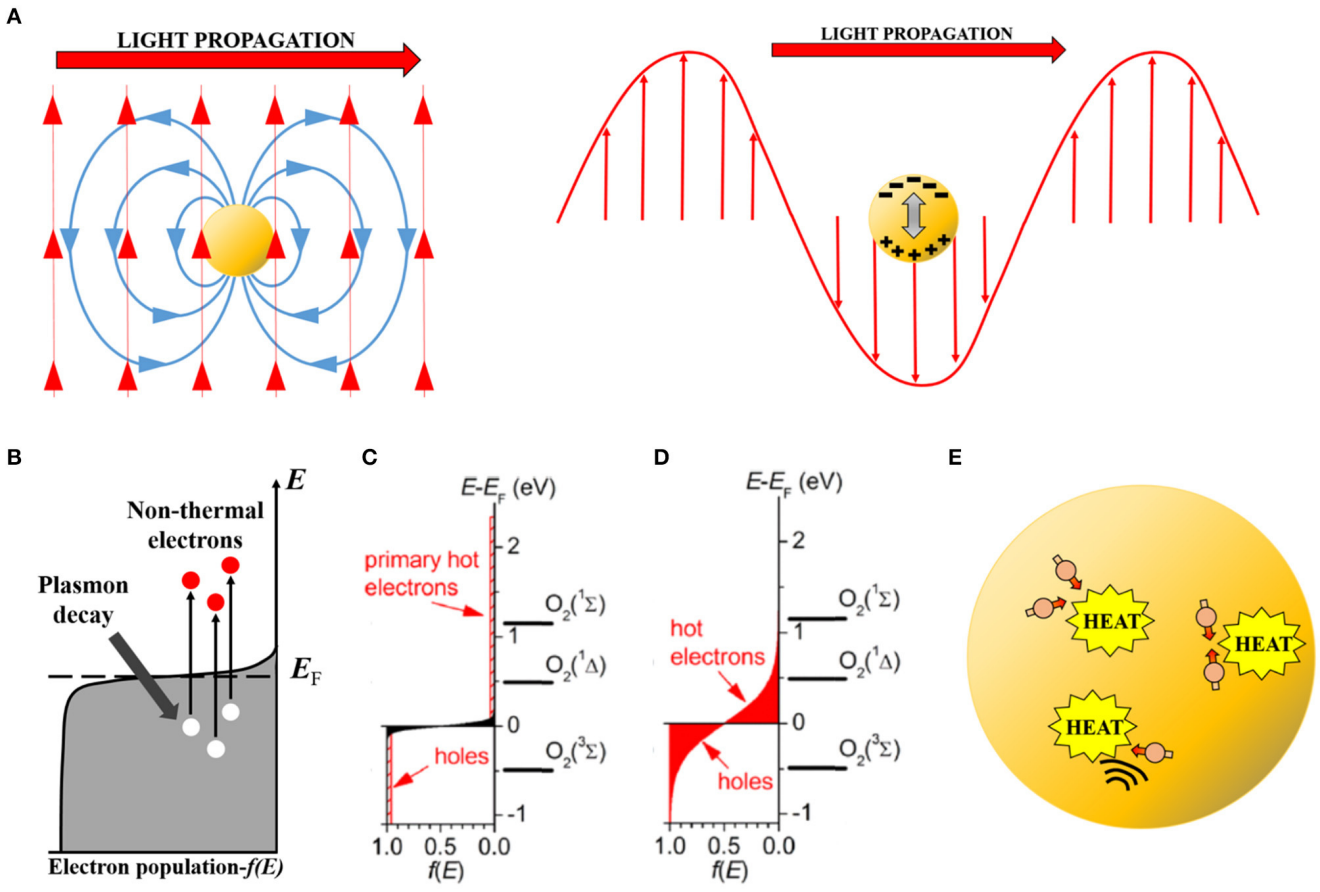
Conceptual scheme showing the different steps in the induction and decay of a plasmon excited in a metal nanoparticle. **(A)** Left, initial electromagnetic induction of an electric field (blue) by the impinging optical electromagnetic field (red). Right, induced internal dipole (plasmon) in the nanoparticle by the optical field (note negative and positive induced charges). **(B)** Non-thermal electron excitation by plasmon decay. Electrons (red circles) are pumped to high energy levels leaving behind holes (white circles). **(C)** Initial population of non-thermal electrons in an intensively excited (by pulsed laser) nanoparticle. Note that electrons reach energies of 2.5 eV and left behind holes of equal energetic magnitude. Molecular oxygen electronic levels have been included on the energy axis for comparison. **(D)** Hot-electron (-hole) populations (red zones) after non-thermal electron (-hole) scattering. Electrons and holes are more abundant than in **(C)** and their energies are still large (up to 1 eV). The first excited state O_2_ (^1^Δ) of molecular oxygen can be efficiently induced under this condition. **(C,D)** Reproduced with permission from Chadwick et al. (2016). **(E)** Hot electrons (dull red circles) and phonons (black waves) scatter, transferring their excitation energy to the nanoparticle lattice which increases its temperature and that of the environment by heat conduction.

The initial step in the metal nanoparticle excitation is the induction of a plasmon resonance (Garcia, 2011; Boulais et al., 2013; Halas, 2019). Metals, including metallic nanoparticles down to very small sizes (<5 nm), present an overlap between the valence and conduction bands: the outer valence electrons do not belong to a particular metal atom, but move around freely (conduction electrons) within the metal. These conduction electrons respond efficiently to outside perturbations, such as electromagnetic fields (i.e., light) (Kim et al., 2017). This fast electronic response occurs at any spatial scale. However, due to their extremely small (nanometric) size, conduction electrons in metallic nanoparticles are perturbed within the whole volume, not just the surface. Under illumination, the oscillating electric field associated to the electromagnetic wave completely “permeates” the nanoparticle (Figure 1A). Initially, the electrons coherently couple to this oscillating electric field and move together as an electronic bunch or cloud. Meanwhile, the atomic nuclei (positively charged) stay fixed in the crystalline lattice. The resulting effect is that of an oscillating electric dipole, the plasmon, in resonance with the impinging light (Figure 1A). The initial plasmon excitation occurs on a time scale of the order of an optical cycle (1–10 fs) (Qiu and Wei, 2014; Amendola et al., 2017). Intuitively, the plasmon “wraps” or “packs” a photon within the nanoparticle, to a size much smaller than the diffraction limit of said photon (100s vs. 10–100 nm).

The coherent electronic oscillation is unstable due to the high probability of electron-electron and electron-phonon interactions (phonons being quantized vibrations of the lattice). After a very brief time (~10 fs) the electronic cloud decouples from the oscillating electric field due to these interactions, and part of the dipole's stored energy is channeled to promote particular electrons to a high-energy (1–10 eV) state (Figure 1B). These high-energy electrons are known, perhaps somewhat counterintuitively, as non-thermal electrons, as their “temperature” (kinetic energy) is much larger than the average temperature within the nanoparticle and they are not in thermal equilibrium with the rest of it (Boulais et al., 2013; Brongersma et al., 2015; Amendola et al., 2017). Depending on the intensity of the exciting light, a smaller or larger subpopulation of conduction electrons will become non-thermal electrons (Figure 1C). These non-thermal electrons have an energy significantly above the Fermi level, which is the energetic level which has an electron occupancy of 50% in a metal (Boulais et al., 2013; Baffou and Quidant, 2014). In other words, the Fermi level represents a threshold energy level for electrons to engage in chemical reactions or leave the particle (ionization).

This initial non-thermal electron population is again unstable to further thermalization by electron-electron scattering (Brongersma et al., 2015; Amendola et al., 2017). The excess energy is swiftly (100–500 fs) redistributed among all the electrons in the particle, leading to a hot thermal Boltzmann distribution (Figure 1D). Depending on the initial energy available, this distribution has a larger or smaller tail of electrons with significantly above-the-average thermal energy. These hot-electrons provide most of the observed chemical reactivity after plasmon excitation. The hot-electron population displays a longer lifetime. Therefore, with their potential chemical energy still large, they can engage in chemical reactions not observed under dark conditions (Kim et al., 2017).

Finally, further electron-electron and, in particular, electron-phonon scattering tend to redistribute heat from the electron population to the whole nanoparticle, including the more massive metal nuclei, increasing its overall temperature (Figure 1E) (Amendola et al., 2017). This thermalizing step usually takes 1–10 ps to complete (Saavedra et al., 2016; Liu J. G. et al., 2018). Depending on the initial energy absorbed, the nanoparticle can heat from fractions of a degree to thousands of degrees. Many biological applications of plasmonic nanoparticles rely on this photothermal effect, but it is beyond the scope of this mini-review. Relevant information on this topic can be found in Qiu and Wei (2014) and Yang et al. (2015).

A few general remarks are pertinent at this point. First, plasmonic properties will vary depending on the particular metal making up the nanoparticle (Kuncewicz et al., 2019). Each metal displays its own plasmonic bands in different spectral regions. Most research has been done with gold nanoparticles, due to their very interesting properties and the possibility to tune the absorption in the NIR biological window (Lv et al., 2015; Yang et al., 2015; Mariano et al., 2018; Sharifi et al., 2019; Zhang et al., 2020). But other metals also show promising features, like silver (Mariano et al., 2018; Seemala et al., 2019), palladium (Long et al., 2013; Liu Y. et al., 2018; Phan et al., 2019), tellurium (Yang et al., 2017), and composite metal-semiconductor nanoparticles (Park et al., 2015; Tatsuma et al., 2017).

Second, there is a very strong influence of the nanoparticle size and shape in the plasmon response, as a consequence of quantum effects arising at such nanometric scales. Therefore, the absorption band can be tuned by just changing the size and/or the shape (Baffou and Quidant, 2014; Yang et al., 2015), and, for larger nanoparticles (>50 nm), or those with non-spherical shapes (nanorods, nanocubes, nanocages, etc.), an electric multipole can be induced under illumination instead of a dipole (Garcia, 2011; Amendola et al., 2017). As a result, changes in the nanoparticle's size result in differences in the photonic response (e.g., favoring photochemistry or photothermal effects, Feng et al., 2019).

Third, different outcomes can be expected if illumination is provided with a continuous wave (cw) source or with a pulsed one, particularly for femtosecond and picosecond lasers. The plasmon excitation process is basically the same in both cases (Figure 1) but under very short pulsed excitation, a significant fraction of the electron population becomes composed of hot-electrons. This alters the optical properties of the nanoparticle, enhancing the optical field close to it (see Nanoplasmas below), and/or favors nanobubble cavitation (photothermal effect) in aqueous solutions or biological systems (Boulais et al., 2013; Besteiro et al., 2019). On the other hand, cw excitation pumps a very small amount of hot-electrons at a time, but does it so at a steady rate. Thus, a continuous photochemistry, which can result in ROS production, will take place (Hogan et al., 2020).

The plasmonic approach to produce energetic charge carriers has some similarities to the photocatalytic process displayed by many semiconductors. Adequately photoexcited semiconductors (e.g., TiO_2_ or ZnO) show electron-hole separation across the band gap, which has been very efficiently employed to promote photochemistry in a diversity of areas (photochemical water splitting, fuel production, etc.), the photogeneration of ROS being a particularly active application in this sense (Mills and Le Hunte, 1997; Serpone and Emeline, 2012). Furthermore, it is a commonplace strategy to synthesized semiconductors along with metal particles to increase the efficiency of these photoprocesses, favoring the separation of the charge carrier (Xu et al., 2019; Zhang et al., 2019b). Recently, a further step has been proposed, by using the metallic particle as the active partner, through its plasmonic excitation, with the semiconductor taking a more passive role on slowing charge carrier recombination and/or taking advantage of its catalytic properties (Fu et al., 2019).

Keeping in mind the similarities between the plasmonic metal excitation and semiconductor photoexcitation, there are some significant differences to be remarked. The plasmonic effect is an initially coherent effect, in which a large electron population reacts to the electromagnetic field provided by the exciting light. In contrast, photoexcitation in semiconductors is considered a (electron-hole)-photon event, independent of other photoexcitations occurring in the semiconductor (it can be argued that very intense photoexcitation by pulsed lasers can produce coherent effects, but this is an effect beyond the current discussion). In plasmonics, an initial high-energy (~10 eV) electron, the result of the plasmon decay, produces several hot-electrons with lower energy (1–5 eV) (Boulais et al., 2013; Brongersma et al., 2015; Amendola et al., 2017). Photoexcitation in semiconductors leads to production of conduction band electrons with energies of 1–3 eV (Mills and Le Hunte, 1997; Xu et al., 2019; Zhang et al., 2019b). Finally, unlike semiconductors, metals do not have a forbidden energy band gap. This translates into faster charge carrier recombination processes in metals as compared to semiconductors. This is an important parameter to consider in regards to the, in general, very small dimensions of plasmonic metal nanoparticles, which favor surface-vs.-volume effects.

## Hot-electron ROS Generation

Once a hot-electron population is generated by plasmon excitation, and before it decays as a thermal wave, there is a time window for these hot-electrons to engage in reactive chemistry with compounds adsorbed at the particle's surface. Two mechanisms have been proposed to explain such reactive chemistry leading to ROS generation: direct hot-electron chemistry and nanoplasmas.

### Hot-Electron Chemistry

Hot-electron chemistry derives from the high chemical potential of these electrons. Interactions at the nanoparticle surface between hot-electrons and adsorbed molecules lead to very efficient redox chemistry. A particular example of relevance for redox biology is the plasmon-driven production of singlet oxygen (^1^O_2_), the first excited state of O_2_. This excited molecule is involved in many redox biological processes (Blázquez-Castro, 2017; Di Mascio et al., 2019) and it is at the mechanistic foundations of PDT (Macia and Heyne, 2015; Chen et al., 2020). As shown in Figures 1C,D, O_2_ energy levels (^3^Σ, ^1^Δ, ^1^Σ) are plotted against the electronic energy levels. Both initial non-thermal electrons, and later hot-electrons have enough energy to directly pump levels ^1^Δ and ^1^Σ of O_2_ molecules. Therefore, ^1^O_2_ is sensitized by energy transfer with these hot-electrons (Chadwick et al., 2016). If a hot-electron directly transfers from the metal surface to occupy the ^1^Δ level, then the radical anion superoxide (•${\text{O}}_{2}^{-}$) will be produced instead of ^1^O_2_. Further reduction of a superoxide anion, either by another hot-electron at the particle surface or through oxidation reaction with a third molecule, will produce hydrogen peroxide (H_2_O_2_), a very relevant ROS in redox biology (Parvez et al., 2018). Nanoplasmas (see below), however, are a source of the very reactive hydroxyl radical (•OH). If no nanoplasma is generated, it seems unlikely that ROS other than singlet oxygen (^1^O_2_) or superoxide (•${\text{O}}_{2}^{-}$) will be produced initially, as they would require more than one electron transfer in sequence (Sies et al., 2017; Kalyanaraman et al., 2018), something improbable given the fast reaction times implicated in the plasmonic affect. Nevertheless, secondary ROS should be produced, as it is well-known the electron avidity of those initials species in order to further reduce themselves toward H_2_O (Kalyanaraman et al., 2018). Furthermore, generation of reactive nitrogen species (RNS) cannot be disregarded at this point, particularly for nanoplasmas (see Nanoplasmas below), as the electron energies involved should be sufficient to initiate molecular nitrogen (N_2_) chemistry. If this turns out to be the case, especially if they can be produced without a nanoplasma, plasmonic excitation could be a novel route by which to produce RNS *in situ* in biological systems (Weidinger and Kozlov, 2015). Of course, this is a simplistic representation of the molecular processes taking place, but is sufficient here to exemplify the kind of interactions that permit the production of ROS after plasmon excitation.

The principal ROS have been successfully produced through plasmon excitation of different metal nanoparticles, such as singlet oxygen (Vankayala et al., 2011, 2013; Gao et al., 2014; Lv et al., 2015; Chadwick et al., 2016), superoxide (Gao et al., 2014), hydrogen peroxide (Wen et al., 2016; Willis et al., 2020), and hydroxyl radical (Gao et al., 2014; Wen et al., 2016). Electrons transferred to oxygen from the nanoparticle can be replenished through oxidation of nearby organic molecules or biomolecules. As metal nanoparticles do not photobleach/oxidize during exposure to ROS, they provide a significant advantage in comparison to classical photosensitizers (Macia and Heyne, 2015).

### Nanoplasmas

An alternative mechanism at work for producing ROS after plasmonic excitation is the creation of a nanoplasma (Boulais et al., 2013). This mechanism only takes place under short pulse excitation (fs-ps) for reasons explained below. Briefly, a nanoplasma occurs when the medium (e.g., water) surrounding the excited nanoparticle ionizes. This plasma breaks down water giving rise to •OH, •H, H_2_O_2_, and also other radicals and reactive molecules (Labouret et al., 2015). Hydrated and solvated electrons are produced too, which are the most powerful reducing agents known (Zilio et al., 2017). The nanoplasma is excited by two mechanisms: electronic emission from the nanoparticle or by plasmon-enhanced electromagnetic breakdown. In the case of electronic emission, the electrons may reach the medium either because they have enough energy to move over the potential surface barrier (non-thermal electrons) or because of thermionic emission (hot-electrons) (Labouret and Palpant, 2016). In plasmon-enhanced breakdown the hot-electrons enhance the optical electric field immediately outside the nanoparticle, decreasing the threshold for plasma breakdown (Boulais et al., 2012). In both cases, quasi-free electrons can couple to the pulsed optical excitation while it lasts, and further drive plasma expansion by inverse bremsstrahlung (Labouret and Palpant, 2016; Zilio et al., 2017). Details of these processes are far beyond the scope of this work and the interested reader is directed to the bibliography for additional information.

Under most experimental situations the laser nanoplasma leads to water superheating and nanobubble evolution. Nanobubble inception requires a threshold electron density of ~10^21^ electrons cm^−3^ (Noack and Vogel, 1999; Vogel et al., 2008). For redox biology applications reaching such a threshold is undesirable, as the goal is to take advantage of the ROS and radicals produced in the nanoplasma and not to create a mechanically disrupting nanobubble (Labouret et al., 2015; Schürmann and Bald, 2016). By carefully choosing the irradiation parameters, it should be possible to obtain adequate electron densities of 10^10^-10^20^ electrons cm^−3^ for biological redox modulation (Vogel et al., 2005; Linz et al., 2015).

## Redox Biology and Plasmonic ROS

**Figure 2:**
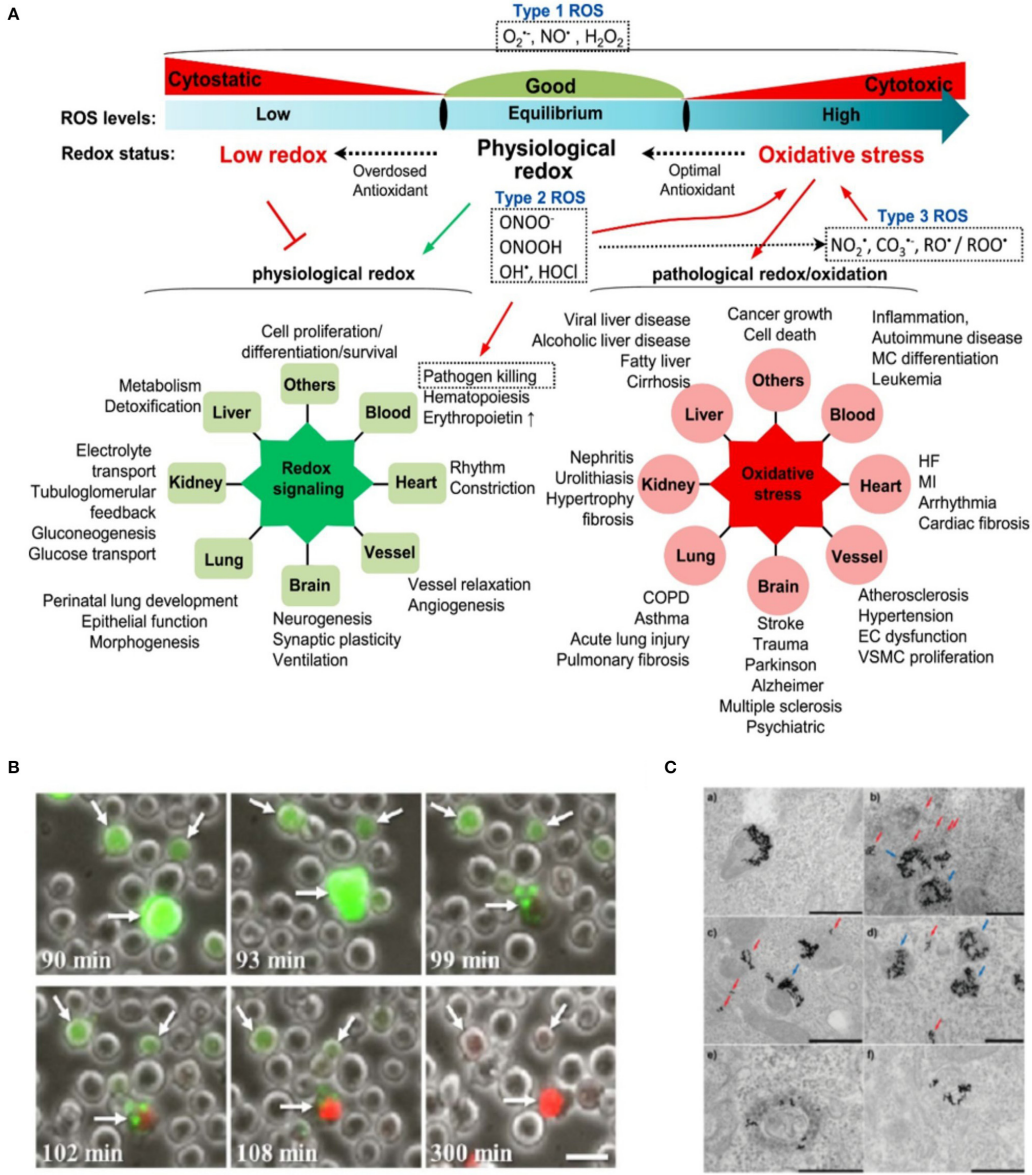
**(A)** Cellular ROS homeostasis and their pathophysiological effects. Cellular ROS levels undergo consistent changes in redox status. Under physiological condition, ROS are maintained at equilibrium levels to facilitate physiological redox signaling (green radial network on the left). Impaired ROS production causes low redox status and suppresses physiological redox signaling. In the case of high ROS status or oxidative stress, excessive ROS would initiate pathological redox signaling and induce cellular damage and various diseases (red radial network on the right). Type 1 ROS is firstly generated and has essential physiological functions. Type 2 ROS and type 3 ROS are subsequently products of Type 1 ROS and play important role in oxidative stress. Reproduced with permission from Zhang et al. (2019a). **(B)** Necrosis in Burkitt lymphoma cells following high intracellular levels of ROS in nanoparticle-targeted cells irradiated by eight 550-nm 50-fs pulses. Numbers at the bottom-left of each frame denote the time elapsed from the moment of irradiation. White arrows point to three representative cells in which excessive ROS (green, dihydro-dichloro-fluorescein ROS probe) have accumulated, promoting cell necrosis (red, propidium iodide vital probe). Scale bar represents 20 μm. Reproduced with permission from Minai et al. (2013). **(C)** Laser inflicted subcellular damage. TEM micrographs of gold nanoparticles in HeLa cells before (a) and after (b–f) cw 514 nm Ar-ion laser irradiation showing examples of the damage to the endosomes; (b–d) dissolution of the membrane of endosomes filled with fewer particles (red arrows) while endosomes filled with more particles tend to remain intact or suffer only minor damage (blue arrows); (e,f) escape of nanoparticles into the cytosol after laser-induced rupture of the endosomal membrane. Laser power density and exposure time: (b) 6 Wcm^−2^, 7 min; (c,f) 20 Wcm^−2^, 1 min; (d) 20 Wcm^−2^, 2 min; (e) 6 Wcm^−2^, 3 min. Scale bars are 500 nm. Reprinted with permission from Krpetić et al. (2010). Copyright 2010 American Chemical Society.

ROS due to plasmon excitation can exert a regulatory or damaging action on biological structures, depending on several parameters, chief among them the ROS dose (Figure 2A). At high doses, biological damage and cell death occurs (PDT). At low doses, more physiological modulation of redox hubs and signaling can be achieved. Some examples of these two scenarios employing plasmonic generation of ROS from illuminated metallic nanoparticles will follow.

### Photodynamic Therapy

At present, the most extended biological application of plasmonic ROS is the destruction of tumoral cells. The formation of ^1^O_2_ by excitation of gold nanorods (AuNRs) with 915 nm NIR light was first demonstrated to effectively kill cancer cells *in vivo* in a B16F0 mouse model of melanoma tumor (Vankayala et al., 2014). The authors showed that the photodynamic effect leading to apoptotic cell death was dependent on the use of very low light doses (<130 mW cm^−2^). Changing the NIR excitation wavelength from 915 to 780 nm induced less effective destruction of solid tumors owing to a combined action of photodynamic and photothermal effects, or just photothermal action.

Experiments using gold nanocages (AuNCs) under NIR one/two-photon irradiation demonstrated a plethora of plasmon-mediated ROS generation mechanisms as previously mentioned (Gao et al., 2014). This study shed light on the advantage of using two-photon vs. one-photon irradiation, by which a striking 6-fold increase in the quantum yield of ^1^O_2_ was achieved. The ability to fine tune intracellular ROS levels paves the way for novel therapeutic strategies (e.g., regeneration) based on a more controlled production of ROS (see Redox Cell Signaling below) (Blázquez-Castro et al., 2012; Carrasco et al., 2015). An example of ROS generation in tumor cells is shown in Figure 2B (Minai et al., 2013). Burkitt lymphoma B cells and epithelial breast cancer cells were targeted by antibody-coated gold nanospheres, then irradiated by a few resonant femtosecond pulses, resulting in significant intracellular ROS. Necrosis was induced between 90 and 300 min after treatment.

The biocompatibility of metal nanoparticles, their adequate bodily clearance and the possibility to easily functionalize them have made them attractive for novel therapeutic strategies. In this regard, the anticancer potential of metal nanoparticles is not restricted to their ability to destroy tumoral cells, but extends to their use as theranostic platforms, integrating diagnosis, treatment and monitoring (Sharma et al., 2015; Sharifi et al., 2019). For example, efficient cancer treatment can be hindered by the particular tumor tissue microenvironment, which can include hypoxia, low pH and relatively high levels of H_2_O_2_. Hypoxia-derived resistance to radiotherapy has been overcome by using Pd@Au bimetallic core-shell nanostructures (TPAN) as a platform to drive plasmon-enhanced robust catalysis of local tumoral H_2_O_2_ under NIR excitation, to promote *in situ* O_2_ production from H_2_O_2_ dismutation in a tumor mouse model (Yang et al., 2019). Under this theranostic approach, core-shell gold nanocage@manganese dioxide (AuNC@MnO_2_, AM) nanoparticles have been proposed as multifunctional platforms to treat and monitor tumors in a breast cancer-bearing mouse model. In this model AM nanoparticles were capable of (i) *in situ* oxygen production by local dismutation of H_2_O_2_ in solid tumors; (ii) multimodal bioimaging; (iii) NIR-dependent generation of additional ROS for oxygen-boosted immunogenic PDT, involving cancer cell destruction and simultaneous anti-tumoral immune response (Liang et al., 2018).

Besides cancer treatment, interesting applications of plasmonic ROS have been reported in other research areas. For instance, plasmonic excitation of copper sulfide nanocrystals can serve as photo-activated sterilizing agents in experimental animal models (Liu et al., 2015). The NIR excitation of these nanoplatforms induced the death of Sertoli cells *in vitro*, as well as upon testicular injection *in vivo* followed by NIR illumination. Another interesting use of gold nanostructures as photodynamic agents has been as antimicrobial tools. Naked gold nanoparticles irradiated with a low-power density Nd:YAG laser efficiently destroyed *E. coli* ATCC 25922 by ^1^O_2_ (Lashkari et al., 2019). In sum, metal nanoparticles and their plasmonic properties can be exploited in a wide range of biomedical applications.

### Redox Cell Signaling

Plasmon-driven PDT has been successfully employed with metal nanoparticles for the last decade, at least in experimental models. The same cannot be said of redox modulation. Milder conditions should be studied under the paradigm of redox biology. For example, it is now proven that gold nanoparticles efficiently sensitize the production of ^1^O_2_ (Gao et al., 2014; Chadwick et al., 2016) and H_2_O_2_ (Gao et al., 2014; Wen et al., 2016; Willis et al., 2020). These ROS are known to act as a redox signaling molecules under the right concentrations and exposure conditions (Ryter and Tyrrell, 1998; Piette, 2015; Carrasco et al., 2016; Blázquez-Castro et al., 2020). Consequently, similar outcomes should be expected for *in vitro* and *in vivo* experiments employing metal nanoparticles and mild plasmonic excitation. Localizing nanoparticles at concrete subcellular sites (e.g., the nucleus, Vankayala et al., 2015) should also permit assessment of intracellular redox signaling (Al-Mehdi et al., 2012; Westberg et al., 2016; Blázquez-Castro et al., 2018).

Another very interesting field of application would be that of redox repair mechanisms. ROS induce rescue and repair responses in biological systems (Epe, 2020). By adequately localizing nanoparticles in particular cellular structures, these mechanisms could be studied. An example is shown in Figure 2C (Krpetić et al., 2010). Authors localized gold nanoparticles to endosomes and selectively damaged these structures under moderate illumination conditions. Cells apparently recovered after the insult and even underwent mitosis for 5 days after the experiment. It has been shown that gold nanoparticles are able to damage DNA bases by redox chemistry under laser exposure (Schürmann and Bald, 2016). Therefore, this could be a complementary approach to study the DNA damage response, a critical detection and repair mechanism for preserving the genetic integrity of a cell (Poetsch, 2020).

## Outlook

Applications of plasmonic effects to redox biology are in their infancy. At present, metal nanoparticles have proven their value as efficient PDT agents. However, experimental research making use of this approach in the area of redox regulation and redox biology is still lacking. It has been our goal to concisely present the fundamentals and advantages of plasmonic ROS generation to the redox biology community, in the hope that, sooner than later, studies with this technique will see the light.

## Author Contributions

AB-C prepared the outline. EC and AB-C wrote and edited the manuscript, and prepared figures. JCS and ÁJ edited and revised the manuscript. All authors approved the manuscript for publication.

## Conflict of Interest

The authors declare that the research was conducted in the absence of any commercial or financial relationships that could be construed as a potential conflict of interest.
